# Development of Therapeutic Competencies in Health Care Students: Qualitative Focus Group Study Using 360-Degree Video and Virtual Reality Technology

**DOI:** 10.2196/75776

**Published:** 2026-02-02

**Authors:** Camilla Lauritzen, Charlotte Reedtz, Kjersti Bergum Kristensen, Vår Mathisen, Eva Therese Næss, Rigmor Furu, Hege Nermo, Rita Jentoft

**Affiliations:** 1 Regional Center for Child and Youth Mental Health Faculty of Health Sciences UiT The Arctic University of Norway Tromso Norway; 2 Department of Health and Care Sciences Faculty of Health Sciences UiT The Arctic University of Norway Tromso Norway; 3 Department of Psychology Faculty of Health Sciences UiT The Arctic University of Norway Tromso Norway; 4 Centre for Health Education Development Faculty of Health Sciences UiT The Arctic University of Norway Tromso Norway; 5 Department of Dentistry Faculty of Health Sciences UiT The Arctic University of Norway Tromso Norway

**Keywords:** health care education, virtual learning resources, virtual reality, therapeutic competence, immersive learning

## Abstract

**Background:**

Therapeutic competence is a critical skill for health care professionals, encompassing communication, interaction, and guidance in vulnerable situations. Virtual reality (VR) and 360-degree video technologies have emerged as innovative tools in health care education, offering immersive and interactive learning experiences. However, there is limited research on their effectiveness in developing therapeutic competencies among health care students.

**Objective:**

This pilot study aimed to explore the feasibility, usability, and perceived educational value of a virtual learning resource using VR and 360-degree video to enhance therapeutic competence in health care students.

**Methods:**

A virtual learning resource was developed, consisting of three modules: (1) a virtual home visit, (2) observation of therapeutic conversations using a 360-degree video, and (3) practice of therapeutic conversations in a simulated environment using VR. The resource was tested with students (n=12) from occupational therapy, psychology, and dentistry programs. Data were collected through focus group interviews after the students completed the modules. Thematic analysis was conducted to identify key themes related to the educational value and learning outcomes of the resource.

**Results:**

The analysis revealed four key themes: (1) active exploration, where students engaged deeply with the material and contextualized theoretical knowledge; (2) observation, which provided practical insights into therapeutic conversations; (3) practice and reflection, which allowed students to refine their skills and build confidence; and (4) translation of theoretical knowledge into practical skills. Students reported that the resource was engaging, immersive, and effective in promoting learning compared to traditional teaching methods. Some students found the VR experience intense but valuable for skill development.

**Conclusions:**

This pilot study demonstrates the feasibility and potential educational value of integrating VR and 360-degree video into health care education. The findings provide preliminary insights into the resource’s ability to enhance therapeutic competence and student engagement. Future research should focus on larger, multi-institutional studies to validate these findings and assess the resource’s impact on measurable learning outcomes.

## Introduction

### Background

Students pursuing health professional education are preparing for professions where they will interact with people in vulnerable life situations. Therefore, it is crucial that these students develop knowledge and skills in interaction, communication, and guidance during their education. In the context of health professions, this can be described as developing therapeutic competencies. Therapeutic competence can be defined as specific skills in conversations and interactions with patients and their families. These skills can be acquired through interactions between students and teachers [1].

Therapeutic competence encompasses a range of skills, including empathy, active listening, and the ability to adapt communication to the patient’s needs and context. It also involves understanding the broader social and familial network of the patient, as well as the impact of the patient’s condition on their family. In this paper, we explore how the development of therapeutic competence can be enhanced through an interactive virtual learning resource, using 360-degree video and virtual reality (VR) technology in teaching. VR and 360-degree videos have been increasingly recognized as effective tools in health care education. These technologies provide immersive and interactive learning experiences, allowing students to practice skills in a safe and controlled environment [2,3].

The learning resource is centered around supporting a family where one of the parents is experiencing mental illness. However, the methodology and therapeutic approach are quite generic and can be applied to several other severe conditions affecting a family, such as parental cancer, injuries, or substance use disorders.

Therapeutic competence also refers to the ability to assess and understand the patient’s overall situation as part of a larger social and familial network. This holistic assessment includes the patient’s roles and responsibilities, which may involve caring for children. An important aspect of this competence is understanding how the patient’s illness affects their family, particularly their children, and adapting health care to the patient’s life situation. Children whose parents experience significant illnesses, such as mental illness or substance use disorders, may require support, information, and follow-up. This perspective on therapeutic responsibilities expands the unit of care provision from a narrow focus on the patient to the wider family and caregiving system and is commonly referred to as a “family focus” in health services [4].

In Norway, health care personnel have a legal obligation to identify whether patients have children, following the revision of the Health Personnel Act in 2010 [5]. This legal provision requires health care personnel providing care to adult patients not only to clarify whether the patient has minor children but also to assess what information or support the family might need. The legislation applies to all illnesses that might impact everyday functioning and parenting, not just mental illnesses [5]. However, extensive national evaluations show that efforts to change practices in hospitals so that all patients with children are assessed and offered support are progressing very slowly [6-9]. It has also been revealed that children are rarely included in conversations with health care personnel when parents are severely ill [10]. The challenges of successfully including children are related to obstacles both within families themselves and within the health care system [10,11]. Internationally, several studies document similar issues. Health care personnel across professions struggle to establish systematic routines that encompass the family situation of the patient [12-14].

Lack of time, competence, and competing tasks are among the reasons why many children do not receive the support and information they need when their parents are severely ill [11]. Part of the explanation may be that health care personnel perceive this work as additional rather than an integrated part of health care [9,11]. Additionally, health care personnel find it challenging to talk to children about difficult topics, such as substance abuse and mental illness in their parents [15].

Acquiring skills in communication, interaction, and guidance for families is thus central to the education of health care personnel. Health care professionals frequently encounter situations that demand therapeutic competence [16]. This requires an understanding of the situation and the ability to interact with the patient in an appropriate manner [17]. Therefore, it is essential that education focuses on developing therapeutic competencies, particularly skills in communication and interaction [16].

### Approaches to Learning Therapeutic Competencies

Emphasizing the active role of students in knowledge development and applying educational approaches that encompass interactivity is an important starting point for developing therapeutic competence [1]. Chi and Wylie [18] describe 4 learning modes, namely passive, active, constructive, and interactive, which were tested in a 5-year project. The results showed that constructive and interactive activities, involving interaction, increased learning outcomes. The students who participated learned significantly more through interactive learning forms compared to traditional 1-way communication from teacher to student [2,3].

Observation can also be an important learning arena for students training to become health care professionals. Studies have shown that health care personnel lack competence in conducting professional conversations with children [4,5], and many are unsure of how to proceed in conversations with families [6,7]. However, by observing family and child conversations, students can gain knowledge about how such conversations can be conducted, although this is challenging to achieve in practice with families in highly vulnerable life situations. Learning through observation is often referred to as the apprenticeship model, which primarily involves the master first explaining or demonstrating how to perform various tasks, followed by beginners observing and trying the tasks themselves [8,9].

Interactive learning approaches and observation should be followed by a component of reflection to optimize the learning of new skills. Reflection is considered an essential component of the learning process, especially when it comes to acquiring new skills in practice [10]. Reflection involves a conscious and systematic assessment of one’s own experiences, actions, and results.

Another aspect to consider is that in the field of children of parents with severe illness, encounters with families where one or both parents face challenges due to illness may reveal children who are not receiving adequate care at home. In such situations, health care professionals may have a legal obligation to report these cases to agencies, such as social services. However, studies have shown that health care personnel largely underreport neglect [11]. To fulfill their reporting responsibilities, it is important that students training to become health care professionals are also trained to recognize risk factors and signs of neglect when interacting with families where serious illness is involved. Reflection promotes a deeper understanding, as it develops the ability to think analytically and solve problems creatively [12]. Additionally, reflection can help build confidence in the role of a health care professional, as one becomes more aware of one’s own competence and potential for development.

Health care personnel report that it can be difficult to ask about sensitive topics, and they are often unsure of what to do with the information they receive [13]. A final aspect that should be emphasized when students are acquiring therapeutic competence and competence in family-focused treatment is the opportunity to practice. To develop skills in asking difficult questions and responding appropriately, it is necessary to obtain practice.

It is ethically challenging to allow students to practice on real patients as interns, especially when it involves complicated life challenges and illness profiles in patients. Practicing through role-play is common, and role-play can be a good way to train professionals for situations that will arise in real life. However, it can be challenging to get all students to participate in role-play in a way that provides learning outcomes because many may find it demanding to be in focus [14]. Furthermore, topics addressed through role-play can trigger negative emotions that may lead the learning process in an undesirable direction [15]. Practicing in simulated and virtual situations can thus be a good alternative. By using virtual learning resources, health science students can practice, among other things, establishing contact and building alliances in an arena where the students’ skills have no consequences for the patient, as they might in an internship situation. Additionally, the virtual situation provides greater opportunities for repeated practice.

### Study Objectives and Research Questions

This pilot study explores the feasibility, usability, and perceived educational value of a virtual learning resource designed to enhance therapeutic competence. The study aims to provide preliminary insights that can inform the development of larger-scale evaluations and more robust research designs in the future.

The research question in the study is “How can a virtual learning resource promote knowledge and skills in therapeutic competencies when interacting with patients who have minor children?”

## Methods

### Overview

The current project aimed to strengthen therapeutic competence in students training to become health professionals by developing virtual and interactive learning resources. The project received funding from the Norwegian Directorate of Higher Education and Skills to develop and test the learning resource. Alongside the development and testing of the resource, an approach inspired by action research was adapted, in the sense that researchers were closely involved in the development and testing process [19]. An important prerequisite was to facilitate systematic development and improvement based on students’ experiences with using the learning resource.

While the study was inspired by action research principles, it does not fully align with the iterative cycles typically associated with action research. Instead, the study focused on piloting and refining the learning resource based on student feedback.

### The Learning Resource: Design, Structure, and Learning Objectives

#### Learning Objectives

To strengthen therapeutic competence development in assessing and offering support to patients who have children, we developed a virtual learning resource for students training to become health care professionals. The objective of the learning resource is to give students and health care personnel insight into family-focused practice and to provide knowledge about how children are affected by their parents’ illness. Furthermore, the learning objective is to teach students what kind of information they need to understand the parents’ illness and its consequences. Additionally, the resource provides an example of how to conduct conversations with parents and children of different ages. The learning resource facilitates an arena for students to practice and reflect on their own performance and encompasses student-active learning forms, observational learning, reflection, and practice. The learning resource consists of 3 modules, which are described below.

#### Modules

##### Module 1

In this module, students go on a virtual home visit to an example family, the Hansen family’s house. This is done as preparatory work through a flipped-classroom approach. Students follow a learning path where they gradually gain more insight into the family’s risk and protective factors. The learning resource provides room for practice and reflection, with interactive tasks and exploration of the case (the family) that encourages participants to assess and develop their own practice. The virtual home visit is easily done using a computer and does not require special equipment. Students walk through the family’s home and acquire knowledge about both the topic area of children as relatives and the example family, the Hansens. This is done by exploring various clickable interactive elements placed in different rooms in the home.

##### Module 2

In this module, students observe conversations with families using 360-degree video and VR headsets. VR refers to a technology that allows the user to interact in a computer-generated environment that mimics reality. VR for skill training has proven to be as effective in teaching as traditional exercises [3]. One of the unique strengths of using VR is that it can provide experiences that would otherwise not be available, such as observation in virtual rooms and practicing specific skills. In this module, the use of VR offers auditory and visual experiences that create a sense of presence in situations that would otherwise not be accessible and can thus enrich the learning process through realistic and engaging simulations.

##### Module 3

**Figure 1:**
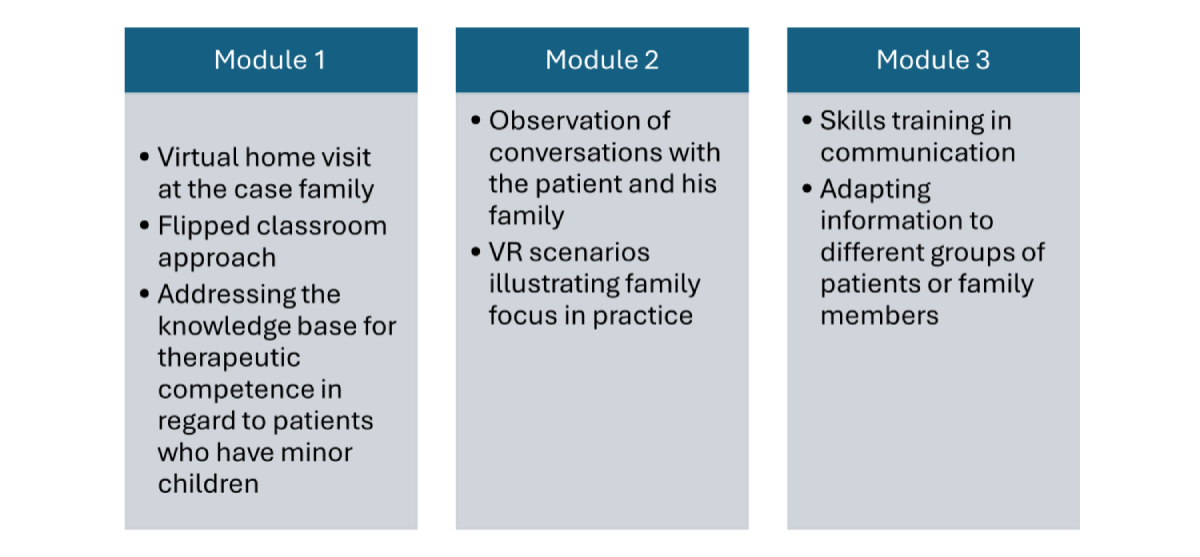
Overview of the modules in the learning resource. VR: virtual reality.

The final module opens opportunities for practicing conversations with the family and the children. With VR headsets on, students sit in the therapy room and interact with the family themselves. The 360-degree videos provide the experience of the family coming into the room to the student as a therapist. There is no dialogue in this module, but the family enters the room and looks directly at the student. It is then up to the student to engage in conversations with the family and the children of different ages. Specific tasks have been created for the module to help the students practice how to express themselves. The exercises for module 3 can be done alone or in groups of students. Figure 1 provides an overview of the entire learning resource.

#### Participants and Procedure in Testing the Learning Resource

The testing of the learning resource was conducted with 2 groups of students recruited from a faculty of health sciences. A total of 12 students participated, including 2 students each from the occupational therapy, psychology, and dentistry programs in each group. The students had limited knowledge of family-focused treatment before the testing. However, they had received some instruction in therapeutic competence and communication, mainly theoretical with elements of role-play. Module 1 was completed at home as preparation, while modules 2 and 3 were completed in a learning lab at the faculty. Based on the experiences from the first group, adjustments were made to the setup for test group 2.

The students used VR headsets for modules 2 and 3. The researchers were present during the entire testing period and took notes consecutively. After finishing the third module, 2 researchers interviewed the students in focus groups. The interviews were transcribed by a transcription service and quality assured by the researchers who conducted the interviews. Analyses of the initial interview revealed minor errors and weaknesses in the learning resource, which were then adjusted before test group 2 tried the resource. The results from test group 2 led to further fine-tuning of the learning resource, and a teacher guide was developed for pedagogical support. The learning resource and guide were then made available in Canvas Commons. Canvas Commons is an integrated content repository for Canvas that lets educators discover, share, and import course materials—like modules, assignments, and quizzes—across courses and institutions. The learning resource is published with open access in line with the principles of open education. We are currently in the process of translating the resource to English, making it freely available for use in international education.

### Analyses

The text data in this study were analyzed thematically. Thematic analysis is based on selected themes by comparing information from all informants in a study [20]. Text data were systematically reviewed by the first author, and the content was sorted into categories by theme. The thematic analysis followed Braun and Clarke’s 6-step framework, which includes familiarization with the data, generating initial codes, searching for themes, reviewing themes, defining and naming themes, and producing the report [21]. Codes were developed inductively based on recurring patterns in the focus group transcripts and were iteratively refined by 2 researchers to ensure consistency and reliability.

The overarching thematic basis for the analysis has been the educational value and learning outcomes of the learning resource. Through the analysis of the interviews, it was identified how the learning resource contributed to learning by (1) exploration; (2) observation; (3) practicing, reflecting, and discussing; and (4) translation of theoretical knowledge into practical skills.

### Ethical Considerations

The project was exempt from ethics review approval by the regional ethical committee, as it does not involve the collection of health information. Privacy considerations have been assessed and approved by SIKT (Norwegian Agency for Shared Services in Education and Research). All informants in the study have been presented with written information material and have signed informed consent forms. The participating students received a small compensation fee for taking part in the testing of the resource.

## Results

### Overview

In the analysis of the focus group interviews, several themes were identified that illustrate how a virtual learning resource can promote knowledge and skills in therapeutic competencies when interacting with patients who have minor children. Overall, the results showed that the learning resource engaged the students and contributed to an active exploration of the learning material, leading to a deeper understanding and better retention of information. Another overarching finding was that the learning resource was perceived by students as a more interactive and engaging learning experience than traditional teaching. Below, we delve into the 4 categories that emerged in the analysis. These were (1) learning through exploration, (2) learning through observation, (3) learning through practicing, reflection, and discussion, and (4) translating what has been learned into practical skills.

### Learning Through Exploration

Students found that the virtual resources facilitated an active exploration process where they could engage directly with the material in an interactive way. Students described how the preparatory work in module 1 allowed them to explore various aspects of the family’s situation, not just by passively reading, but by investigating and assessing the family situation and viewing the situation from different perspectives. The interactive exploration led to a deeper understanding of the topic and made it easier to remember what they had learned compared to traditional methods, for example, receiving a lecture with Microsoft PowerPoint on the subject matter. The dialogue below illustrates this:

Did it make it a bit easier to grasp the health legislation this way, compared to having something presented on a PowerPoint? You’re nodding, yes.

Interviewer

Yes, that’s a very good example, with the legislation... I hate when it’s introduced on a slide. It’s something I can’t manage to learn when it comes on PowerPoint, whereas here I could almost remember the paragraph word by word afterward.

Student

I found it very helpful when the legislation was very connected to the situations and the examples. They were tailored to the situations you had either gone through or were going to go through, so it was a bit like: “okay, that applies here, and that applies there.” It made much more sense. Because when you’re reading it in a textbook, it’s like: “Yes, it probably applies there.” But when you could see why, it was way better.

Student 2

So, it was easier to remember it?

Interviewer

Yes, really.

Student 2

Speaking of what he said about retaining the information, because in our educational program, we constantly ask for clinical examples. Put it into context, give us something to attach it to, and don’t just list an 80-page PowerPoint with legislation. We can’t grasp that. But receiving something concrete, I think, can be used in many other contexts too, and it is so much easier to learn when you have something to attach it to.

Student 1

In both focus groups, there was broad agreement that being able to explore the topic themselves, in this case through a virtual home visit, was perceived as promoting learning. It made abstract theory about the consequences of being a child of parents with various life challenges, as well as training in health legislation, more concrete and comprehensible.

Some students found it somewhat intense to work with the topic in this way. The quote below illustrates this:

Before I came here, I had an expectation that it would be interesting and educational. I’m generally interested in therapeutic competence, communication forms, and such things. But I didn’t think it would be as all-encompassing as it actually was. When I did module 1 on the PC at home yesterday, I even found it more educational than a PowerPoint. So, now in VR mode, I almost felt my brain boiling, because there was so much info. I started thinking like: “Who should I look at? Should I look at her or him? Who am I most curious about in this family?” And that’s a good thing, but yes, very intensive learning. So, it’s a great resource, but I noticed I was a bit caught off guard. I didn’t know it would be so many stimuli and impressions.

Student

This finding shows that it can also be overwhelming to work with such topics in VR, and that teachers therefore must consider that various reactions may arise in the student group. Discomfort can also be a source of development and lead to students experiencing a high learning outcome. Being able to explore the case on their own and become familiar with the challenges the example family had was perceived as relevant and educational.

Another student expressed this as follows:

I think that often when we do case-based learning, the information that is important in the case is pointed out to you. But in this exploration, you get a lot of information to assess yourself. With being in such an interactive resource, you get the opportunity to choose for yourself what you want to focus your attention on. You practice much more on noticing the important things, compared to when you just get presented with something. When you have to look for it yourself, you also practice focusing your attention. So, I thought that was very cool.

Student

In the conversation about exploring the topic area on their own using the interactive, virtual home visit in module 1, the students reported that it had a high educational value. The students said that it resembled reality. When students are in internships or start practicing as healthcare personnel after their education is finished, they don't necessarily know what the patients’ or families’ issues are. They have to use their developing therapeutic competence and knowledge to assess risks and problem areas to work on moving forward. The overarching finding in this category was that the learning resource was perceived as a very practice-oriented and effective way to learn these skills.

### Learning Through Observation

The students reported that observing therapeutic conversations and communication between the therapist, patient, and the patient’s family was very educational. The observations allowed them to see the acquired theory applied in practice. Students discussed how they observed different communication styles in conversations with children versus adults and how the therapist adapted their language and methods according to the client’s age and level of understanding. This provided students with a practical understanding of how to tailor therapeutic techniques to different client groups.

Observing these conversations can be used as a starting point for skills training and give us the opportunity to practice therapeutic competence aimed at children, adolescents, adults, and older adults. It allows us to see what we need to do as therapists to bring out the best possible information about their life situation, and in what way we can use ourselves as tools to acquire information.

Student

Another student expressed it as follows:

It was very educational because you get to see a real situation of how both the therapist and those coming to the therapist express themselves, and how their body language is. In my case, I learn a lot by viewing examples, like in an internship, by watching how my supervisor does it, and this was similar.

Student

The overarching finding from this category showed that students experienced high learning outcomes from observing a conversation between a therapist and a family.

### Learning Through Practicing, Reflection, and Discussion

VR offers a unique opportunity not only to observe but also to actively engage through simulated therapeutic sessions. This leads to reflection and discussion, which are crucial for learning and developing therapeutic competence. Participants appreciated the opportunity to discuss and reflect on their actions and decisions in the VR scenarios, as shown in the dialogue below:

How did you find reflecting along the way? Useful or not useful?

Interviewer

I generally think just seeing that you have different ways of perceiving things, and that someone can make you aware of something you absolutely weren’t aware of. We discovered that we disagreed on how we perceived the situation, and I think it’s great to see different viewpoints.

Student 1

Yes, and it triggers a much longer thought process too.

Student 2

I thought it was very educational, even though I felt tired at the end.

Student 3

I thought the questions were good. The questions allowed us to reflect on several possibilities and perspectives instead of giving us the answer immediately.

Student 4

This excerpt from one of the focus group interviews highlights the value of questions that stimulate reflection. This helps them understand and internalize the learning material better.

Another important pedagogical value that emerged in the focus groups was the opportunities the VR resource provided for practice. The dialogue below illustrates this:

I noticed that for me to practice my therapeutic competence, I need a certain calm. Because I noticed that I didn’t quite know where to start at first, because I was stressed by them [the family] staring at me. It takes practice, I think, because you have to be able to be in a certain situation several times to be able to do it well. So, I felt that I got into it the more I practiced.

Student 1

To get into a kind of flow. I started to think about my body language eventually. After a while, I started to think: “How am I sitting now, actually? Is it okay to sit like this?” But at first, I couldn’t even think about what to say.

Student 2

The overarching result in this category is that students experienced good learning outcomes from trying out, practicing, reflecting, and discussing with each other using this learning resource.

### Translating What Has Been Learned Into Practical Skills

Finally, the students discussed how they can apply what they have learned through VR in their professional practice. This includes applying knowledge and skills in real therapeutic situations, such as handling sensitive topics with clients or adapting communication styles based on the client’s needs and situation. Students reflected on how they can use insights from the learning resource to improve their practice, especially in how they interact with clients and handle complex and sensitive issues. The dialogue below illustrates this:

So, what do you think about conducting such a conversation now? What would it be like?

Interviewer

One of the things I’m a bit nervous about is exactly that. But it feels a bit easier now. I feel like I know more.

Student

Students also reported that they have become more aware of what the term therapeutic competence entails. They also mentioned that they had become more attentive to the whole family and the importance of involving relatives. The dialogue below illustrates this:

I’m also a bit curious about what you now consider to be therapeutic competence. What do you think now? Is it clear to you what it is?

Interviewer

It’s a whole bag of things, with listening and hearing, tips, tricks, what works, what doesn’t, reflecting, getting them to become aware of things, and not just like: “Pull yourself together, get well!”

Student 1

I became much more aware that maybe we should try to involve families more often.

Student 2

The overarching result in this category is that, in terms of translating theoretical knowledge into actual therapeutic competence, the students had positive learning outcomes by using the learning resource.

## Discussion

### Principal Findings

This pilot study explored the feasibility, usability, and perceived educational value of a virtual learning resource designed to enhance therapeutic competence in healthcare students. The findings suggest that the resource has the potential to promote active exploration, observation, and practice in a safe and controlled environment. Students reported that the resource was engaging and effective in promoting learning compared to traditional teaching methods. The study's findings align with existing literature on the benefits of interactive and immersive learning approaches in healthcare education [2,18].

The results also showed that the learning resource provided students with the opportunity to observe and practice therapeutic conversations in a safe environment. This aligns with the need to develop skills in adapting communication and interaction to different patient groups. These skills are crucial for responsible professional practice. VR technology offers a unique opportunity to learn through observing simulated, yet realistic, therapeutic conversations. VR technology can simulate complex interactions with patients and their families, providing students with some experience, yet avoiding the ethical concerns that may arise from practicing directly on patients. This is particularly relevant in education, where direct practice with real patients can be challenging or difficult to achieve, such as in the early stages of educating health professionals. Compared to role-play, where students themselves play the different roles, using films of relevant scenarios where students practice being a therapist who observes, listens, and interacts through VR technology appears more direct and thus professionally relevant than inventing thoughts, feelings, and behaviors in people they play themselves. Furthermore, the students developed new concepts and understanding through reflection and discussion of what they had observed in the scenarios. They could relate legislation and therapeutic techniques to practical situations, showing that they moved from concrete experience to abstract conceptualization [22]. Translating what they had observed into concrete skills seemed, therefore, easier using this learning resource.

The learning resource is designed for students to reflect on their observations, often referred to as reflective observation [22]. Using reflection as a key component in the learning process, the opportunity to reflect on their observations was provided, and the students appreciated this option. The students found that the questions in the learning resource stimulated deeper thinking, reflection, and discussion, which is crucial for developing a deeper understanding and ability to adapt to new challenges. By discussing how the VR experience allowed them to see theory applied in practice, they felt they gained a deeper understanding of the material. Reflection on what they observed, such as communication styles and the therapist's adaptation to the client's age, thus helped strengthen their learning.

The learning resource gave the students an opportunity to practice and refine their interaction skills in a controlled environment before applying them in real situations. Practice is essential for developing skills. Students reported that they experienced increased confidence and flow in communication after repeated exercises in the VR environment. This underscores the importance of practice for achieving mastery and confidence in therapeutic practice. Trying out new skills in a safe environment can be essential for active experimentation. The students reported that practice in VR helped them achieve flow and confidence in therapeutic conversations, which is a critical part of the learning process. Practicing in VR was perceived as activating and engaging, and the students found that the resource provided a deeper understanding as well as promoted the development of skills.

Translating theoretical knowledge into practical skills can be difficult to achieve with traditional learning methods. Students reported that the VR experience provided a more vivid and realistic understanding of the subject matter, contributing to more effective learning. Students described the VR experience as particularly valuable because it allowed them to “step into” scenarios relevant to their field of study. This feeling of being part of the scenario contributed to a more authentic and engaging learning experience. The students felt that visualization contributed to a better understanding of complex subject matter. The learning resource created a unique opportunity to explore, observe, and interact in complex situations in a way that text or images cannot. In light of Kolb’s experiential learning cycle [22], one can say that the learning resource provides a concrete experience that is essential to start the learning cycle. Students could see body language, hear tone of voice, and observe interactions in a controlled but realistic environment. This helped them understand complex subject matter and integrate theoretical and practical aspects of the therapeutic competence they will need in practice. Through exploration, observation, reflection, and discussion, the learning resource thus contributes to the acquisition of experience, laying the foundation for the development of therapeutic competence.

VR-based practice offers significant advantages for developing foundational therapeutic communication skills, such as active listening, empathy, and adapting communication to different client groups. The immersive nature of VR allows students to observe and practice interactions in realistic scenarios, which can enhance their confidence and readiness for real-world practice [23]. However, certain aspects of authentic therapeutic interaction, such as emotional attunement, relational pacing, and the influence of cultural and familial contexts, may be more challenging to replicate in a virtual environment. These elements often require nuanced, real-time adjustments that are best developed through direct interaction with patients and guided supervision. To address these limitations, the learning resource was complemented with structured reflection activities that allowed students to explore the subtleties of therapeutic relationships in greater depth.

Therapeutic communication is a complex skill that develops progressively throughout a health care professional’s education and career [1,16]. For students in the early stages of their training, foundational skills, such as active listening, empathy, and basic conversational techniques, are critical [18,22]. The virtual learning resource aligns with these developmental needs by providing a safe and controlled environment for students to practice these foundational skills. Unlike residents or more experienced practitioners, students may have limited exposure to real-life therapeutic interactions, making simulated environments particularly valuable for bridging the gap between theoretical knowledge and practical application. Future studies could explore how the resource might be adapted for learners at different stages of their professional development, including residents and practicing clinicians, to address more advanced aspects of therapeutic communication.

Equity and fairness are critical considerations in the implementation of VR-based learning environments. Learners come from diverse cultural, linguistic, and socioeconomic backgrounds, which can influence their communication styles and access to technology. Similarly, patients and families bring unique cultural and contextual factors that shape their communication needs and expectations. To support equitable outcomes, the learning resource could be adapted to include scenarios that reflect a wide range of cultural and familial contexts. Additionally, structured reflection activities and supervisory guidance could help learners develop cultural competence and inclusive communication practices. Future iterations of the resource should also consider accessibility features, such as subtitles or alternative formats, to ensure that all learners can fully engage with the material.

### Practical Implications

Although VR can have significant educational benefits, several challenges must be addressed to maximize educational value and learning outcomes. It is important to consider and assess the ethical aspects of using VR in education, because simulations of sensitive topics can have unforeseen consequences. Teachers using such learning resources should be prepared for emotional reactions and have a plan for how to handle such reactions. As with all teaching on sensitive topics that are perceived as challenging for students, it requires educators to help students navigate these challenges by providing support and guidance throughout the learning process and through supervision.

User-friendliness and technical support are important prerequisites when applying virtual resources to teaching situations [23]. Participants in the focus groups reported the need for technical support and guidance when using VR, emphasizing the importance of accessible and effective technical assistance. Another challenge is the cost and availability of VR technology. Although the prices of VR equipment have fallen in recent years, costs can still be a barrier for many educational institutions. Furthermore, effective use of VR requires appropriate software and updates, which can also incur significant costs. It also requires some logistics to ensure that equipment is charged and updated with new software so that it can be easily used.

It is also important to consider how VR can be integrated and systematically implemented in educational programs to maximize learning outcomes. To ensure that VR technology becomes an integrated part of educational programs, educational institutions must consider both infrastructural and pedagogical adjustments. This requires anchoring with management, investment in necessary technology, organization of training, use, and guidance at the institution. By using VR technology, however, one can achieve more targeted competence development. In many health professions, VR scenarios can be tailored to meet the need for developing specific clinical and therapeutic skills.

### Limitations

This pilot study involved a small sample size (n=12) and a single-case context, which limits the generalizability of the findings. The study was designed to provide preliminary insights into the feasibility and perceived educational value of the virtual learning resource, rather than to draw definitive conclusions about its effectiveness. Future research should include larger, more diverse student groups and multi-institutional studies to validate the findings and assess the resource’s impact on measurable learning outcomes. Additionally, comparative studies with control groups using traditional teaching methods would provide a more robust evaluation of the resource’s relative advantages and limitations.

While the study was inspired by action research principles, it did not fully adhere to the iterative cycles typically associated with action research. Instead, the study focused on piloting and refining the learning resource based on student feedback. Future research could adopt a more comprehensive action research approach, incorporating multiple iterations and cycles of feedback to further enhance the resource and its implementation.

### Conclusion

This pilot study demonstrates the feasibility and potential educational value of integrating VR and 360-degree video into health care education. The participating students experienced that the virtual learning resource promoted the learning of therapeutic competence. Interactive learning, practice, and reflection were important elements that contributed to the students’ development of skills necessary to meet the demands of health professions. The findings provide preliminary insights into the resource's ability to enhance therapeutic competence and student engagement. Future research should focus on larger, multi-institutional studies to validate these findings and assess the resource’s impact on measurable learning outcomes.

## Abbreviations

SIKT: Norwegian Agency for Shared Services in Education and Research
VR: virtual reality

## Data Availability

The datasets generated or analyzed during this study are not publicly available because they consist of qualitative interview data that cannot be adequately anonymized, and written consent to store the data in an open repository was not obtained prior to the study.
